# Prescription of Anti-Spike Monoclonal Antibodies in COVID-19 Patients with Resistant SARS-CoV-2 Variants in Italy

**DOI:** 10.3390/pathogens11080823

**Published:** 2022-07-22

**Authors:** Daniele Focosi, Marco Tuccori

**Affiliations:** 1 North-Western Tuscany Blood Bank, Pisa University Hospital, 56124 Pisa, Italy; 2 Division of Pharmacovigilance, Pisa University Hospital, 56124 Pisa, Italy; marco.tuccori@gmail.com

**Keywords:** SARS-CoV-2, COVID-19, monoclonal antibodies, bamlanivimab, etesevimab, casirivimab, imdevimab, sotrovimab, tixagevimab, cilgavimab, Ronapreve™, Evusheld™

## Abstract

Anti-Spike monoclonal antibodies have been considered a promising approach to COVID-19 therapy. Unfortunately, the advent of resistant lineages jeopardized their effectiveness and prompted limitations in their clinical use. Change in the dominant variant can be fast to such an extent that, in the absence of timely medical education, prescribers can keep using these drugs for relatively long periods even in patients with resistant variants. Therefore, many patients could have been exposed to drugs with unlikely benefits and probable risks. We show here that about 20% of bamlanivimab+etesevimab, 30% of casirivimab+imdevimab, and 30% of sotrovimab courses were administered in Italy during periods in which a fully resistant variant was dominant. Additionally, for monoclonal antibody cocktails, the vast majority of usage occurred against variants for which one of the mAbs within the cocktail was ineffective. Given the high costs of these drugs and their potential side effects, it would be important to consider a frequent review of the appropriateness of these drugs and timely communication when the benefit/risk balance is no longer favorable.

## 1. Introduction

Anti-SARS-CoV-2 Spike monoclonal antibody (mAb), either as monotherapy or as a cocktail of mAbs, was a pillar of COVID-19 outpatient treatments in the second half of 2021, when sensitive variants of concern (VOCs) ravaged the globe. As for any other European Medicine Agency (EMA) member nations, the Italian Drug Agency (Agenzia Italiana del Farmaco, AIFA) promptly granted mAb authorization. Despite growing evidence of treatment-emergent resistance [1,2], the use of mAbs continued even after oral antivirals became available, particularly in frail patients with contraindications to the use of small molecule antivirals. Unfortunately, the advent of the Omicron VOC led to a loss of effectiveness for most of the authorized mAbs [3,4,5,6].

While randomized controlled trials (RCT) that led to product authorization by regulatory authorities were led at the time of sensitive VOCs, in vitro data have consistently shown that the Omicron BA.1 VOC was notoriously resistant to both bamlanivimab and etesevimab [7,8,9,10,11,12,13,14,15,16,17] and casirivimab plus imdevimab [7,8,9,10,11,12,13,14,18,19], while the Omicron BA.2 VOC is notoriously resistant to sotrovimab [20,21,22,23,24,25].

The tixagevimab plus cilgavimab mAb cocktail, approved to date only for pre-exposure prophylaxis, represents a special case: while the tixagevimab component has been ineffective against any Omicron sublineage so far (BA.1 [7,10,11,12,13,16,26], BA.2 [26,27], and BA.4/BA.5 [26,28]), the cilgavimab component is ineffective against BA.1 [7,10,11,12,13,16] and BA.4/BA.5 [26,28] but has preserved efficacy against BA.2 [26,27]. A similar scenario occurred for both the other two mAb cocktails. Against the Beta and Gamma VOC, casirivimab lost in vitro activity [29,30,31] while imdevimab [30,31,32,33] preserved it. Against the Delta VOC, bamlanivimab lost in vitro activity [34,35,36,37,38,39], but etesevimab preserved it [37]. Under these scenarios, the chances for immune escape much grow, theoretically as when delivering a monotherapy [1,40].

Total changes in dominant VOCs were relatively fast (once every 2–3 months), questioning whether continued medical education was timely in place to avoid hazardous and expensive prescriptions. While only a few patients had viral genome sequencing performed to confirm definitive resistance, statistical inferences can be made moving from prevalences detected by epidemiological surveillance. In this article, we investigated the use of anti-SARS-CoV-2 Spike mAbs in patients affected by resistant SARS-CoV-2 VOCs in Italy.

## 2. Materials and Methods

We collected prevalence data of different SARS-CoV-2 variants in Italy from the reports of the flash surveys led by the Italian Istituto Superiore di Sanità (ISS) from 25 May 2021 to 27 May 2022, available at https://www.iss.it/web/guest/cov19-cosa-fa-iss-varianti (accessed on 15 June 2022). Briefly, according to the indications from the Italian Ministry of Health, every 15 days about 2000 newly diagnosed COVID-19 patients with positive RT-PCR are further processed with whole-genome sequencing (WGS), and the sequences are attributed to PANGOLIN phylogeny using a dedicated web portal (ICOGEN).

We collected absolute prescription counts for each authorized anti-Spike mAb (or mAb cocktail) from the Italian Drug Agency (Agenzia Italiana del Farmaco, AIFA) web portal, available at https://www.aifa.gov.it (accessed on 15 June 2022). The monitoring registry was established according to a Ministry of Health decree issued on 6 February 2021. Briefly, every 7 days (every 15 days since May 2022) a report is issued showing the cumulative count of prescriptions for each anti-Spike mAb. In order to keep the graph at a weekly sampling, 15-day counts were manually estimated as 7-day counts, assuming a homogenous within-period daily distribution. In order to account for the high standard deviation related to the small sample size, we considered two different thresholds of SARS-CoV-2 VOC diffusion in the population (50% and 99%) to identify periods in which the prescriptions of the mAbs could have likely occurred in patients harboring a resistant VOC. 

## 3. Results

**Figure 1:**
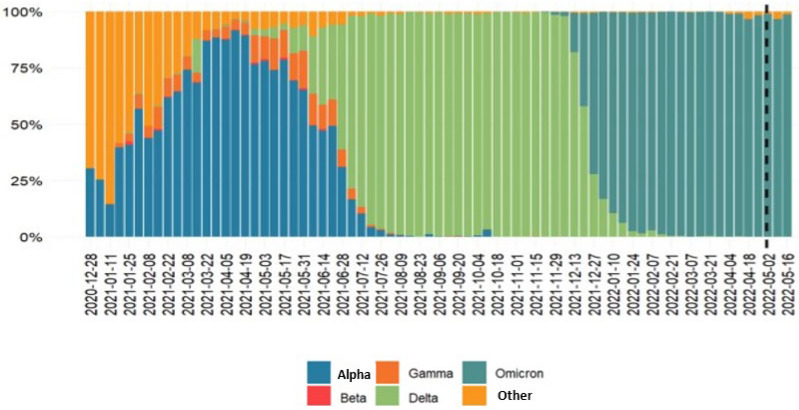
Prevalence of various SARS-CoV-2 variants in Italy since December 2020 according to ISS flash surveys reports.

Figure 1 shows the changes in the prevalence of SARS-CoV-2 VOC in time in Italy according to flash survey reports. Based on established thresholds of SARS-CoV2 variant diffusion, we identified 20 June 2021 as the date in which the Delta variant accounted for 50% of the infections and 15 August 2021 as the date in which the Delta variant accounted for 99% the of infections. The dates of Omicron BA.1 and BA.2 50% diffusions were 20 December 2021 and 20 March 2022, respectively. The dates of Omicron BA.1 and BA.2 99% diffusions were 31 January 2022 and 4 May 2022, respectively.

The bamlanivimab and etesevimab cocktail was authorized by the AIFA on 17 March 2021. The casirivimab and imdevimab cocktail was authorized by the AIFA on 26 November 2021, while sotrovimab was authorized on 23 December 2021. Finally, the tixagevimab plus cilgavimab cocktail was authorized by the AIFA on 25 March 2022. At the time of writing, neither the EMA nor the AIFA have withdrawn authorization for any of these mAbs.

**Figure 2:**
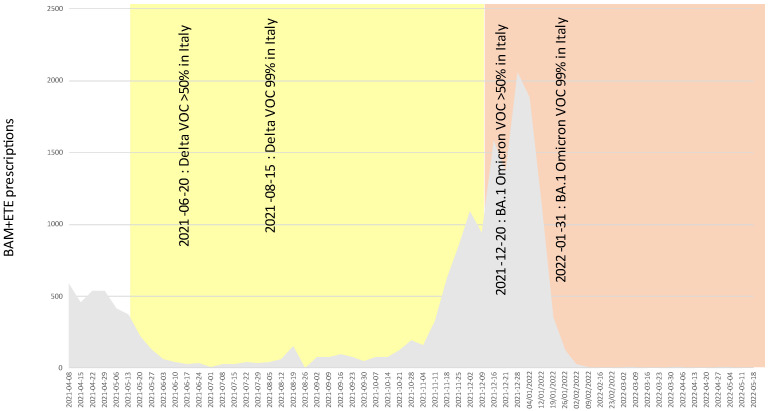
Absolute prescription counts of bamlanivimab + etesevimab (BAM + ETE) in Italy. Areas with yellow shading indicate partially inappropriate usage (bamlanivimab being ineffective against VOC Delta). Areas with red shading indicate likely inappropriate usage (prevalence of resistant VOC BA.1 higher than 50% according to ISS flash surveys). As a reference, dates of FDA advice or withdrawals are reported.

**Figure 3:**
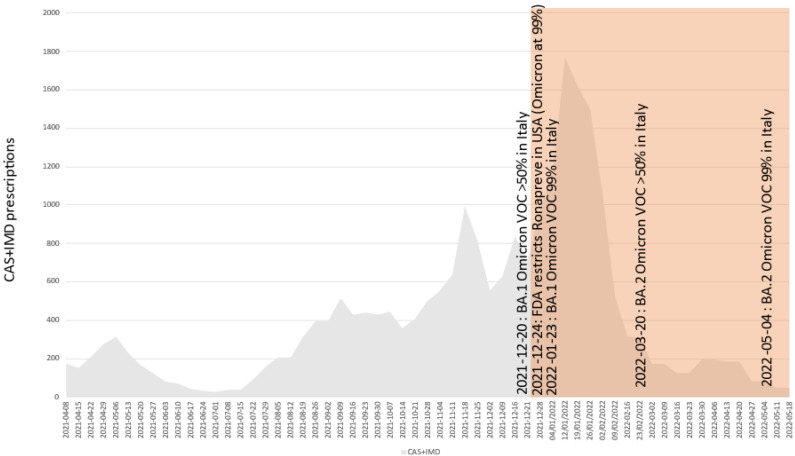
Absolute prescription counts of casirivimab + imdevimab (CAS + IMD) in Italy. Areas with red shading indicate likely inappropriate usage (prevalence of resistant variant(s) higher than 50% according to ISS flash surveys). As a reference, dates of FDA advice or withdrawals are reported.

**Figure 4:**
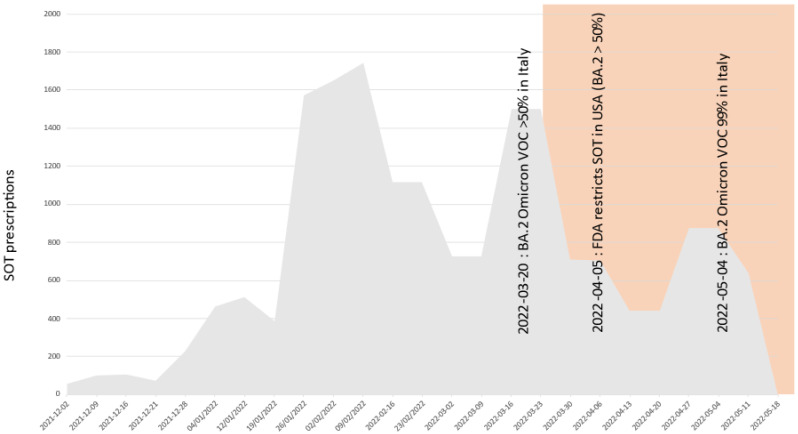
Absolute prescription counts of sotrovimab (SOT) in Italy. Areas with red shading indicate likely inappropriate usage (prevalence of resistant BA.2 VOC higher than 50% according to ISS flash surveys). As a reference, dates of FDA advice or withdrawals are reported.

**Figure 5:**
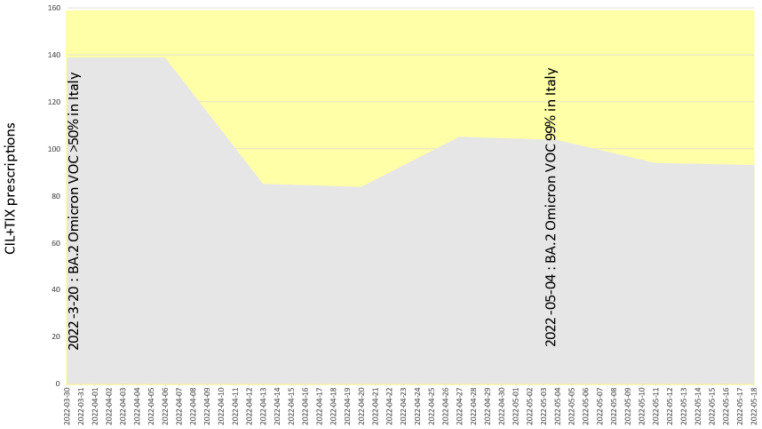
Absolute prescription counts of tixagevimab + cilgavimab (TIX + CIL) in Italy. The entire area is yellow-shaded because tixagevimab was not effective at neutralizing against Omicron BA.1 or BA.2, while cilgavimab was effective only against BA.2.

Figure 2 shows that in Italy about 60% of the bamlanivimab plus etesevimab cocktail was prescribed while Delta was dominating (hence making bamlanivimab delivery inappropriate), and that about 20% was prescribed at the time the fully resistant VOC BA.1 was dominating. Figure 3 shows that about 30% of the casirivimab plus imdevimab cocktail was prescribed after the fully resistant VOC BA.1 became dominant, and that (albeit largely reduced) prescriptions are still happening while BA.2 is dominating. Figure 4 shows that about 30% of sotrovimab was prescribed after the fully resistant VOC BA.2 became dominant, and usage is still ongoing and undisturbed at the time of writing. Figure 5 shows the usage of the tixagevimab plus cilgavimab cocktail, which occurred during the BA.2 wave, against which tixagevimab was ineffective.

## 4. Discussion

Our analysis showed that a relevant proportion of anti-SARS-CoV-2 Spike mAb prescriptions in Italy could have occurred in patients with resistant disease. Given that national regulatory authorities within Europe usually act in alignment with recommendations by the EMA, we cannot exclude that, in the absence of any communication by the EMA, the situation could be similar in many other European countries. To the best of our knowledge, no other study investigating this issue has been published so far using data from other European countries. In this regard, the policy of access to COVID-19 treatments deployed by the FDA appeared suitable to limit the use of these drugs in patients with probable resistant disease [4,5].

**Figure 6:**
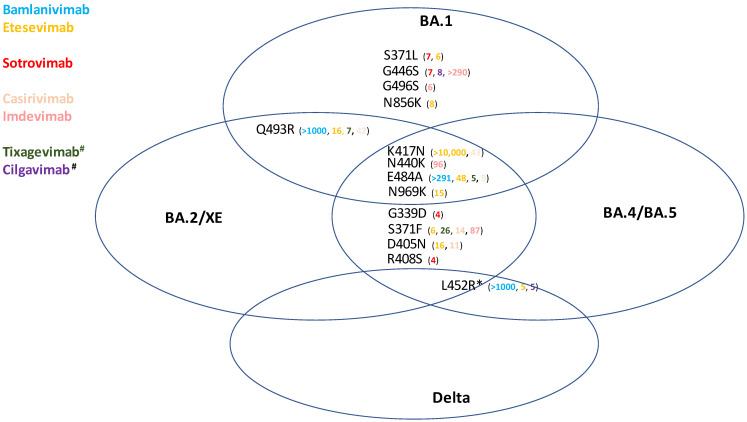
Mutational patterns of Spike proteins predicting resistance to SARS-CoV-2 VOCs. Values represent the geometric mean neutralizing antibody titer fold-reduction caused by specific mutations. Sourced from https://covdb.stanford.edu/page/susceptibility-data/ (accessed on 15 June 2022). ^#^ approved for prophylaxis only; * L452occur in all BA.4/BA.5 lineages, but only in a minority of BA.2 sublineages (e.g., BA.2.11 and BA.2.35).

Despite the time shift of a couple of weeks between sample collection and flash survey reporting, this explanation is clearly not enough to account for the large share of mAbs used inappropriately. The alternate reasons for this delay in the adjustment of prescription habits should be analyzed in detail. We believe that the lack of RCTs to support in vitro evidence plays a prominent role in driving prescribers’ attitudes: e.g., most clinicians tend not to read or underestimate in vitro evidence, which was promptly released during the pandemic. Nowadays, resistance can be highly predicted on the basis of Spike mutational patterns observed in vitro (Figure 6). Our hypothesis is substantiated by publications reporting usage in Italy of bamlanivimab against Gamma [41] despite notorious in vitro resistance [29,30,31,42], or the AIFA-sponsored MANTICO trial, authorized by the Ethical Committee of the National Institute of Infectious Diseases (INMI), which reported usage of casirivimab plus imdevimab against BA.1 [43].

An alternative hypothesis is that, when frail patients cannot tolerate small-chemical antivirals (which have retained efficacy against all Omicron sublineages), available mAbs can apparently offer a tolerable option, particularly when other supposedly effective mAbs (bebtelovimab [24]) are not available in Europe yet. In this regard, we note that COVID-19 convalescent plasma (CCP) collected from vaccinees retains efficacy against all Omicron sublineages [44], but we regret that, despite being encouraged by both CDC/IDSA and ECIL-9 guidelines, CCP collection and usage has been almost abandoned in Europe. CCP is also superior to mAbs in terms of the risk of immune escape [2].

A third possibility is pressure from regional administrators to use large stocks that have been ordered and would otherwise remain unused. In this regard, fine-tuning of orders and pay-by-performance mechanisms could mitigate the risk.

Although mAbs generally have a favorable safety profile, their prescription in patients with resistant COVID-19 disease comes with several drawbacks: under constrained resources (as it commonly occurs in pandemic settings), wastage of money invariably translates into less investment in alternative and possibly effective therapeutics. These considerations also apply to mAb cocktails where one of the ingredients is not effective.

In summary, the timely medical update of the prescribers should be implemented in situations in which the epidemiological scenario of the disease is so dynamic. In these scenarios, regulatory agencies should consider closer monitoring of the epidemic patterns of the resistant variants of the disease and react with timely actions and communications.

## Data Availability

The data are freely available at the sources provided in the Methods section.

## Abbreviations

VOC: variant of concern; ISS: Istituto Superiore di Sanità; AIFA: Agenzia Italiana del Farmaco.
